# Individual Postprandial Glycemic Responses to Meal Types by Different Carbohydrate Levels and Their Associations with Glycemic Variability Using Continuous Glucose Monitoring

**DOI:** 10.3390/nu15163571

**Published:** 2023-08-13

**Authors:** Jiwoo Song, Tae Jung Oh, YoonJu Song

**Affiliations:** 1 Department of Food Science & Nutrition, The Catholic University of Korea, Bucheon 14662, Republic of Korea; sjwjulie@naver.com; 2 Department of Internal Medicine, Seoul National University Bundang Hospital, Seongnam 13620, Republic of Korea; ohtjmd@gmail.com; 3 Department of Internal Medicine, Seoul National University College of Medicine, Seoul 03080, Republic of Korea

**Keywords:** postprandial glycemic response, mixed meal, dietary carbohydrate, glycemic variability, glycemic control

## Abstract

This study aimed to investigate individual postprandial glycemic responses (PPGRs) to meal types with varying carbohydrate levels and examine their associations with 14-day glycemic variability using continuous glucose monitoring (CGM) in young adults. In a two-week intervention study with 34 participants connected to CGM, four meal types and glucose 75 g were tested. PPGRs were recorded for up to 2 h with a 15 min interval after meals. Data-driven cluster analysis was used to group individual PPGRs for each meal type, and correlation analysis was performed of 14-day glycemic variability and control with related factors. Participants had a mean age of 22.5 years, with 22.8% being male. Four meal types were chosen according to carbohydrate levels. The mean glucose excursion for all meal types, except the fruit bowl, exhibited a similar curve with attenuation. Individuals classified as high responders for each meal type exhibited sustained peak glucose levels for a longer duration compared to low responders, especially in meals with carbohydrate contents above 50%. A meal with 45% carbohydrate content showed no correlation with either 14-day glycemic variability or control. Understanding the glycemic response to carbohydrate-rich meals and adopting a meal-based approach when planning diets are crucial to improving glycemic variability and control.

## 1. Introduction

Maintaining blood glucose levels in the normal range or as close to normal as possible is crucial in the prevention and management of metabolic diseases such as obesity, metabolic syndrome, and type 2 diabetes. Postprandial hyperglycemia is considered a cardiovascular risk factor, even in nondiabetic subjects [1]. A meta-analysis of 38 prospective studies confirmed a strong association of the 2 h postchallenge blood glucose level with fatal and nonfatal cardiovascular events in apparently healthy individuals without diabetes [2]. One-hour postprandial glucose levels were also reported to be associated with increased cardio-ankle vascular index (CAVI) values and the degree of arterial stiffness in Japanese nondiabetic subjects [3] and were associated with an increased risk of incident diabetes mellitus in Korean adults with normal glucose tolerance at baseline [4]. 

Dietary carbohydrates are a major determinant of the postprandial glycemic response (PPGR). Carbohydrate metabolism is fundamental, ensuring a constant and essential energy supply to living cells. The relationship of dietary carbohydrates with cardiovascular disease and disorders of carbohydrate metabolism has been a subject of prolonged discussion [5]. The glycemic index (GI) of carbohydrate-containing foods has played a substantial role in predicting postprandial glycemic response for nearly 40 years. However, it has limited applicability in mixed meals, and several studies have reported the utility of the GI in applying it to mixed meals. There have been reports that the mean glucose responses of mixed meals were similar to those of the individual foods [6], and a calculated meal GI model created by adjusting the protein and fat content of meals has been proposed in order to predict the glycemic impact of mixed meals [7]. However, the clinical utility of the GI for mixed meals remains questioned due to the high variability of individual responses.

By developing and using continuous glucose monitoring (CGM), glycemic variability can be accurately evaluated in studies. According to a study that tracked PPGR to test meals in an 800-person cohort using CGM, the PPGR to identical meals is reproducible within the same person, but the PPGRs of different people to the same meal show high variability [8]. Another large-scale study to assess human postprandial response to foods using CGM revealed that meal composition has greater effects on PPGR than genetic influence. The meal context, including meal timing, has higher effects than expected [9]. Indeed, several studies using CGM reported that meal distribution, such as restricting carbohydrates in the morning, improved glycemic variability [10] or reduced mean glucose and fasting blood glucose [11]. 

Despite other factors influencing PPGR, meal composition is the primary determinant of PPGR and variability in people. The Korean diet is traditionally high in carbohydrates and low in fat and is mainly composed of white rice with plenty of plant-based dishes. As white rice is a core food, its contribution to total energy is considerable, although an overall decrease has been observed in young populations [12]. Since Asian staples are dominantly high-GI foods, it is worth noting a study wherein a typical Asian diet replacing staples with low-GI sources improved 24 h glycemic excursion and variability parameters [13]. Another study also reported that a healthy dietary pattern differentiated only by major sources of starch in meals improved glycemic variability in low-GI sources [14].

However, most studies have examined the effect of certain types of diets, such as a low-glycemic-load diet or a high-protein diet, on glycemic variability using a cross-over design [15,16]. Few studies have focused on individual postprandial glycemic responses to various meal types with varying carbohydrate levels. Understanding the individual glycemic response to meal types with high carbohydrate content is crucial for maintaining blood glucose levels within the normal range and planning meals in daily life. With this background, this study aimed to investigate individual glycemic responses to four common meals with different carbohydrate levels and explore their associations with 14-day glycemic variability from CGM in apparently healthy young adults without diabetes.

## 2. Materials and Methods

### 2.1. Study Design and Participants

This was a prospective two-week interventional study to test individuals’ postprandial glycemic response to standardized meals. The participants were recruited between March 2022 and May 2022 online or through flyers describing the study. Inclusion criteria were healthy young adults aged 18–38 who could engage in 2 weeks of intervention and wear a continuous glucose monitoring device. The exclusion criteria included skin complaints or taking medication for metabolic diseases or being scheduled for treatment with strong magnetic or electromagnetic radiation (e.g., X-ray, MRI, CT, etc.) during the intervention. Of the 46 applicants, 4 were excluded through screening and 6 due to scheduling problems. After screening, 2 were excluded due to health problems and COVID-19, and 1 could not have meal type B due to a health problem (Figure S1).

**Figure 1:**
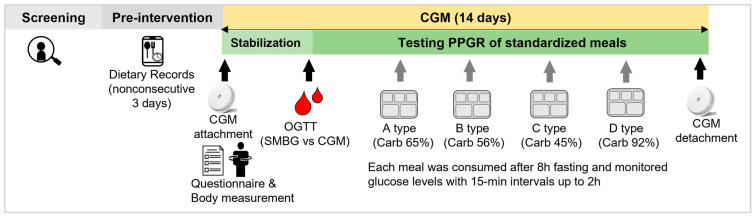
The study design testing individual postprandial glycemic responses to four meal types and their associations with 14-day glycemic variability in 34 young adults. CGM, continuous glucose monitoring; PPGR, postprandial glycemic response; OGTT, oral glucose tolerance test; SMBG, self-monitoring blood glucose.

The key scheme of the study design is presented in Figure 1. Participants were instructed to record nonconsecutive 3 days of dietary records in the pre-intervention week. At the first visit, participants underwent baseline examination, which included general questionnaires and body measurements, and were connected to continuous glucose monitoring (CGM). At least 2 days after the first visit, participants performed an oral glucose tolerance test (OGTT) of glucose 75 g and compared blood glucose level CGM to self-monitoring blood glucose (SMBG) for validation. Blood samples for SMBG were taken by pricking the fingertip with a lancing device, and the blood glucose level was immediately measured using a glucose meter (CareSens N Premier, i-SENS Inc., Seoul, Korea). Afterward, participants were requested to visit in a fasting state after 8 h of fasting. Four meals were provided by staff according to the preference of the participants. The meals were prepared by the staff, and the food was weighed using an electronic scale (KB-2000, A&D Co., Seoul, Korea). Participants visited on a desired date and were asked to consume a meal within 30 min and stay 2 h after the meal to record blood glucose levels at 15 min intervals. When they completed the last meal, the participants were allowed to remove the CGM. All participants were informed about the study protocol and consented to participation by signing the consent form. They received a reward after their participation was completed. This study was approved by the Institutional Review Board of the Catholic University of Korea (No. 1040395-202203-07) and registered with the Clinical Research Information Service (CRIS) (KCT0007158), which is an online registration system for clinical trials and clinical research conducted in Korea and is affiliated with the WHO International Clinical Trials Registry Platform (ICTRP).

### 2.2. Standardized Test Meals

The four meal types were meals commonly consumed by young adults in Korea, characterized by moderate to high carbohydrate contents: (A) a rice-based meal, (B) a sandwich meal, (C) a chicken salad, and (D) a fruit bowl. The food ingredients and nutrient composition of each meal are presented in Table 1.

The rice-based meal was composed of white rice with variety of seasoned vegetables (called bibimbap) and soybean paste soup (Type A), the sandwich meal was composed of ham and cheese sandwich with soybean milk (Type B), the chicken salad meal was composed of chicken breast with various fresh vegetables (Type C), and the fruit bowl meal was composed of assorted fresh fruits (Type D). The energy intake of meals ranged from 345.8 to 673.2 kcal, with carbohydrate contents from 59.3 g to 94.0 g. Four meals were classified by carbohydrate levels as percentage of energy: 65% for type A, 56% for type B, 45% for type C, and 92% for type D. 

Nutrient composition for each meal was calculated using the Korean Food Composition Database 9.3 from the Rural Development Administration of Korea (Korean Food Composition Table (revision 9.3). Rural Development Administration. Korea. 2021). The glycemic index (GI) for each meal was obtained from the international tables, and dietary glycemic load was calculated by multiplying the food item GI by the amount of food carbohydrates consumed and dividing by meal’s total carbohydrates [17,18]. 

The dietary glycemic load (DGL) of the mixed rice meal was 63.7, which is higher compared to other meal types. On the other hand, the DGL of the fruit bowl was 46.7, the lowest among meal types, primarily due to its high fiber content from whole fruits.

### 2.3. Postprandial Glycemic Response and Grouping

The individual postprandial glycemic response was measured in a fasting state after at least 8 h of fasting before meal and at 15 min intervals for 2 h after meal using CGM. The AUC (area under the curve) was calculated for all the areas under the blood glucose response curve down to a blood glucose concentration of 0 using the trapezoidal formula. The incremental AUC (iAUC) calculated the area under the blood glucose response curve down to fasting blood glucose [19]. Each individual was tested with four different meals as well as glucose 75 g on separate days, and the average fasting blood glucose was calculated as the mean of fasting blood glucose levels obtained on five different days. To check the clinical accuracy of blood glucose between CGM and SBGM, Clarke Error Grid Analysis was conducted. Based on a total of 34 paired CGM-SBGM measurements, the Clarke Error Grid Analysis showed 100% of glucose values falling into clinically acceptable error zones A and B (Figure S2). 

In order to classify individual PPGRs for each meal type, a data-driven cluster analysis was conducted using postprandial glucose levels measured at 10 different time points, including fasting blood glucose. Two distinct clusters emerged within each meal type based on the similarity of their postprandial glucose levels. These clusters were labeled as a “High-response” group, with relatively high PPGR, and a “Low-response” group, with relatively low PPGR, for ease of discussion.

### 2.4. CGM Measurement

Subjects were connected to CGM device (Freestyle libre, Abbott Diabetes Care, Alameda, CA, USA) during the two-week study period. The sensor was attached to the triceps of the participant and affixed with an adhesive bandage and was removed on the day of finishing PPGRs with the last test meal. The subjects were instructed to record the postprandial glucose levels for each test meal before meal and at 15 min intervals after meal for up to 2 h. All procedures were monitored by a staff member. 

The CGM outcome metrics included mean glucose and coefficient of variation (CV), time in target range (70–180 mg/dL), time above the range, and time below the range. Glycemic control was defined as mean glucose, and glycemic variability was defined as CV during the CGM active period.

### 2.5. Body Measurement

Body composition, height, and waist circumference were measured to determine the basic characteristics of the participants. Height was measured using an extensometer (DS-102; Dong Sahn Jenix Co., Ltd., Seoul, Republic of Korea). Body composition was measured using a machine via bioelectrical impedance analysis (InBody 370S; Inbody Co., Seoul, Republic of Korea) to determine weight, muscle mass, fat mass, and percent of body fat. BMI was calculated using the formula “BMI = kg/m^2^”. Waist circumference was measured using a flexible tape measure at the midpoint between the lowest rib and the uppermost pelvic bone. 

### 2.6. Dietary Assessment

To evaluate participants’ usual intake, participants were asked to record their daily diets on 3 nonconsecutive days (2 weekdays and 1 weekend) in week before the intervention. Participants were asked to record the time and place of their meals, food, and intake in as much detail as possible, and dietary records were collected through a mobile app. Meals were classified into breakfast snacks, breakfast, morning snacks, lunch, afternoon snacks, dinner, and late-night snacks. All dietary data and nutrient intakes were analyzed using the Diet Evaluation System, an online dietary survey and nutrition assessment program.

### 2.7. Other Variables

Participants were asked to complete a questionnaire about general characteristics, including sex; age; and lifestyle factors, including alcohol consumption, current smoking, and physical activity. Participants who consumed more than the standard amount (5 cans of beer for men and 3 cans for women) of alcohol at least once a month were classified as drinkers, and those who smoked every day were classified as current smokers. Physical activity was classified into low-intensity, moderate-intensity, and high-intensity exercise based on the International Physical Activity Questionnaire short form (IPAQ) [20].

### 2.8. Statistical Analysis

All continuous variables are presented as the mean ± standard deviation, and all categorical variables are presented as a number and percentage (n, (%)). Categorical variables were analyzed using the Chi-square test, and continuous variables were analyzed using the Wilcoxon rank-sum test due to small sample size. 

To compare distributions of individual postprandial glucose levels according to meal types, the box-and-whisker plot was used. The bottom and top of the box are the lower and upper quartiles, and the whiskers are lines outside the box that go to the minimum or maximum. The outliers are defined as values outside 1.5 times the interquartile range. To characterize individual postprandial glycemic responses, cluster analysis (PROC FASTCLUS) using K-mean algorithms was applied for 10-time-point glucose levels according to each meal type. Based on analyses with larger numbers of cluster solutions and our earlier work [21], the suitable number was determined. All meal types derived two distinctive clusters, later labeled with descriptive names. To investigate the factors associated with 14-day glycemic control and variability, correlation analysis was performed with all variables, including basic and body composition, dietary intake from dietary records, postprandial blood glucose at 2 h, and iAUC for each meal type. Due to small sample size, Spearman correlation coefficients were presented after adjusting for sex. Using Clarke Error Grid Analysis, a scatter plot was generated, and the grid was inserted using Microsoft Excel [22]. All statistical data were tested using SAS 9.4 software (SAS Institute Inc., Cary, NC, USA), and statistical significance was set at *p* < 0.05.

## 3. Results

### 3.1. Participant Characteristics

The general characteristics of the 34 participants are presented in Table 2. The mean age of the subjects was 22.5 years, and the percentage of men was 26%. The mean BMI was 22.8 kg/m^2^. The proportions of current smokers, subject to physical activity levels, showed a significantly different distribution according to sex (*p* = 0.0033, *p* = 0.0464). The muscle mass (kg) and waist circumference (cm) were significantly higher in men than in women (33.0 ± 5.1 vs. 20.8 ± 2.8, *p* < 0.0001; 83.5 ± 9.5 vs. 71.6 ± 8.5, *p* = 0.0025), and the body fat (%) was significantly higher in women than in men (32.8 ± 7.1 vs. 21.3 ± 8.3, *p* = 0.0034). There were no significant differences in other variables, as well as nutrient intakes, between men and women. 

### 3.2. Mean Postprandial Glycemic Response According to Meal Type 

**Figure 2:**
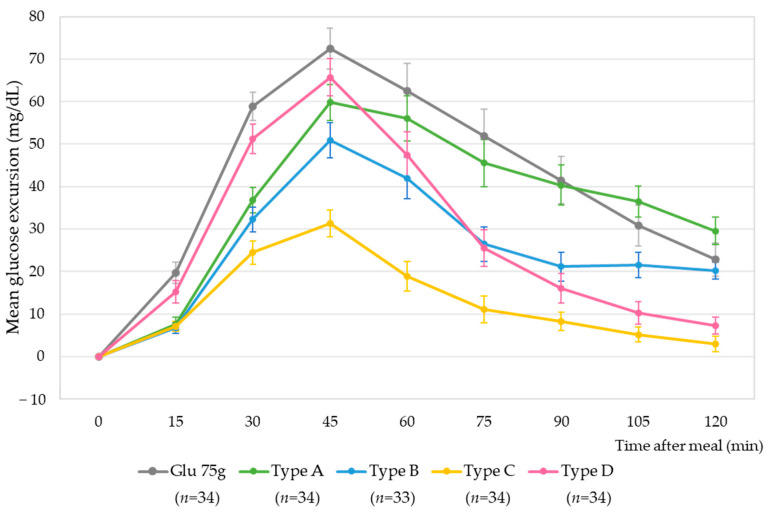
Mean postprandial glucose excursions from fasting blood glucose and up to 2 h after meals in 34 young adults. Error bars represent means ± SE.

The mean postprandial glucose excursions according to meal types from fasting up to 2 h after meals are presented in Figure 2. All test meals were served as the first meal on a separate day. The average meal duration ranged from 15 min to 22 min by meal type, and there was no difference in fullness (Table S1). The glucose 75 g exhibited considerably higher glucose excursions than all meal types. The peak of glucose excursions occurred 45 min after the meal, gradually attenuating over 2 h following the meal. The rice-based meal had a similar curve to 75 g of glucose with attenuated glucose excursion. The sandwich meal showed lower glucose excursion than the rice-based meal but higher than that of the chicken salad, as expected. The chicken salad meal showed modest glucose excursion. On the other hand, the fruit bowl displayed a similar increase in glucose excursions up to 45 min after the meal, followed by a considerable decrease. However, the glucose levels did not return to fasting levels, even after 2 h.

### 3.3. Individual Postprandial Glycemic Responses According to Meal Type

**Figure 3:**
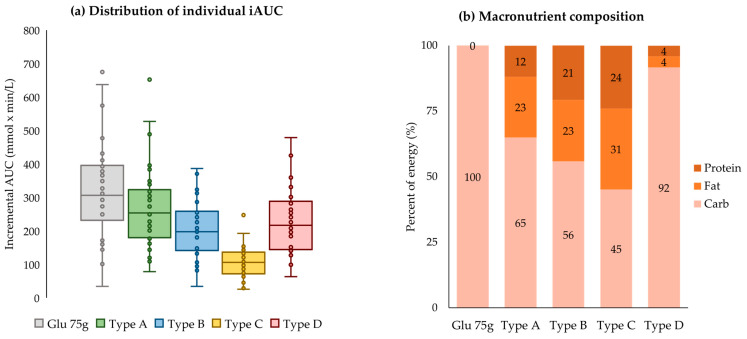
Individual postprandial responses to meal type and nutrient composition.

Figure 3 presents the distribution of the individual incremental AUC and macronutrient composition by each meal type. The box plot visualizes the distribution of individual postprandial glycemic responses (iAUC) for each meal type, allowing for easy comparison between the first and third quartiles. The box plot revealed that the range of iAUC was highest for glucose 75 g (249.8 to 393.5 mmol × min/L), followed by the rice-based meal (181.3 to 314.8), the sandwich (147.6 to 255.2), and the chicken salad (75.9 to 134.9). This order corresponds to the carbohydrate contents of each meal type, except the fruit bowl, with the rice-based meal having the highest carbohydrate intake as a percentage of energy at 65%, followed by 56% in the sandwich and 45% in the chicken salad. On the other hand, the range of iAUC in the fruit bowl (145.4 to 288.5) was slightly lower compared with that in the rice-based meal. The box plot also displays outliers beyond 1.5 times the interquartile range. Each meal type showed outliers at some time points, which indicates that some individuals respond very heavily to the same meal.

### 3.4. High- vs. Low-Response Groups for Each Meal Type

**Figure 4:**
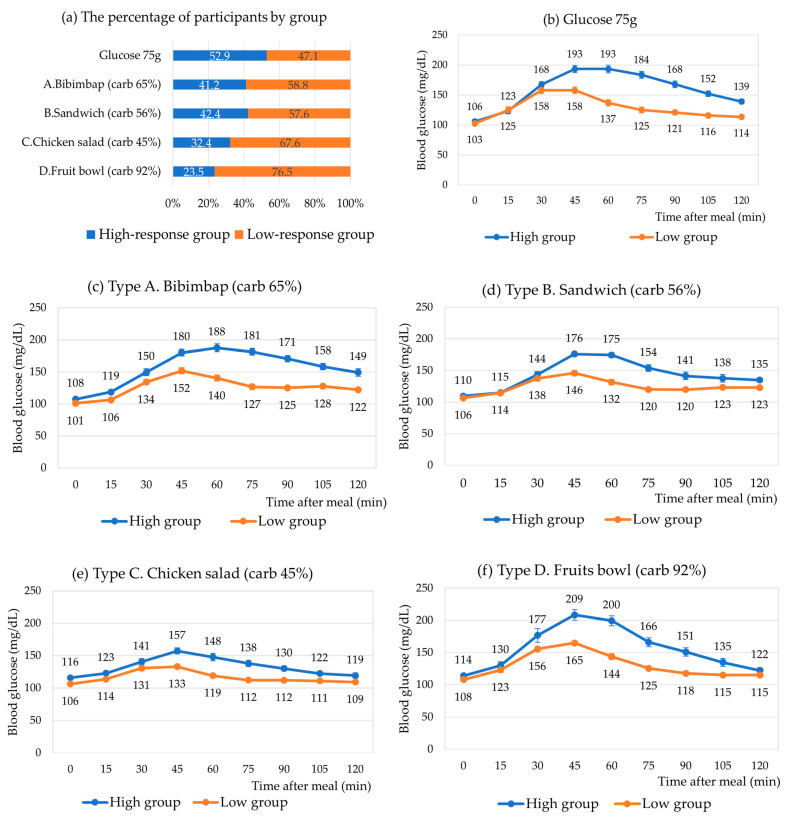
The comparison of postprandial glycemic response to meal types in high- or low-response groups, classified by cluster analysis.

Based on the similarity of their 10-time-point glucose levels after meals, all meal types, including glucose 75 g, were categorized into high responders and low responders. The proportions of the two distinct groups for each meal type are presented in Figure 4. Approximately half of the subjects were classified as high responders to glucose 75 g, while the remaining were categorized as low responders. Among participants, 41.2%, 42.4%, and 32.4% were identified as high responders to the rice-based, sandwich, and chicken salad meals, respectively. Only 23.5% of subjects were classified as high responders to the fruit bowl meal.

Overall, high responders across all meal types exhibited higher postprandial glucose levels, particularly from 45 min to 120 min after the meal. In the case of the rice-based meal, the glucose levels in low responders peaked 45 min after the meal and gradually declined. In contrast, high responders exhibited a marked increase in glucose levels starting 45 min after the meal and sustained until 75 min and then attenuated. The difference in PPGRs between the two groups was more pronounced in the rice-based meal compared to the sandwich and chicken salad meals. In the case of the fruit bowl meal, a small percentage of subjects (23.5%) exhibited a particularly strong response, with the glucose levels 45 min after the meal being higher in high responders compared to other meal types.

### 3.5. Factors Associated with 14-Day Glycemic Variability and Control

Table 3 represents the correlation analysis of factors associated with 14-day glycemic variability and control. Age and body composition at baseline did not correlate with either 14-day glycemic variability or control. The average daily intake of carbohydrates, protein, and fat, obtained from a 3-day dietary record, was not correlated with either 14-day glycemic variability or control.

However, postprandial glucose levels at 2 h (PP2) and incremental AUC (iAUC) for each meal type were found to be significantly correlated with both 14-day glycemic variability and glycemic control after being adjusted for sex. Specifically, 14-day glycemic control showed significant correlations with PP2 for all meal types, while 14-day glycemic variability showed significant correlations with iAUC for all meal types except the chicken salad meal (carb 45%). Neither average fasting blood glucose nor 14-day mean glucose showed any correlation with 14-day glycemic variability. 

## 4. Discussion

In this two-week intervention study, we investigated individual glycemic responses to four mixed meals with varying carbohydrate contents in young adults using CGM. Our finding revealed that individual glycemic responses were categorized into two response groups, even if the carbohydrate level of the meal was lowered. We observed that individuals classified as high responders for each meal type exhibited sustained peak glucose levels for a longer duration compared to low responders, especially in meals with carbohydrate contents greater than 50%. Additionally, mean glucose excursion curves for meal types were influenced by the carbohydrate proportions of the meals, and a meal with a carbohydrate content of 45% showed no correlation with either 14-day glycemic variability or glycemic control. 

The main feature of this study was how individuals’ glycemic responses were affected by four mixed meals with varying carbohydrate contents. A Korean diet typically consists of white rice as the main staple, accompanied by various side dishes. It is worth noting that white rice consumption has been reported to increase the risks of type 2 diabetes in the Asian population [23]. In our study, we examined the glycemic response to a rice-based meal consisting of white rice and a variety of vegetables. While the mean glucose excursion curve was slightly attenuated in a rice-based meal compared to glucose 75 g, individuals classified as high responders in the rice-based meal exhibited nearly the same postprandial glucose levels as those classified as high responders in glucose 75 g. 

Furthermore, a study investigating Mediterranean-style eating patterns plus a major source of starch (low vs. high GI) revealed that a diet based on high-GI foods with major starch increased glycemic variability despite adherence to an overall healthy pattern, compared to a diet based on low-GI foods [14]. Another study also focused on healthy ingredients in Asian staples and demonstrated improved glycemic control and variability [13]. These studies appear to offer a feasible dietary strategy for healthy adults; however, considering the individual glycemic response, it may not be necessary for everyone to replace staples with low-GI foods but only for those identified as high responders.

One of the interesting findings was that the individual glycemic responses were categorized into two response groups, even if the carbohydrate level of the meal was lowered. This finding is in line with the finding that meal carbohydrate contents correlated well with PPGRs in some people (carbohydrate-sensitive), while some people showed little relationship between meal carbohydrates and PPGR (carbohydrate-insensitive) [8]. Recent studies reported that a personalized postprandial-targeting diet showed improved glucose control and metabolic health in adults with newly diagnosed type 2 diabetes [24] or prediabetes [25] compared to the commonly recommended uniform Mediterranean diet. These studies emphasized a personalized diet based on individual PPGR using a machine-learning algorithm, where there are different sets of foods for individuals. Unfortunately, due to high costs and technologies, this personalized diet cannot be widely used. However, knowing one’s own PPGRs to typical meals with high carbohydrate contents can be a useful tool for healthy meal planning. 

In our evaluation, we also considered the PPGR for a sandwich meal, which represents a bread-based meal option, and a chicken salad, which serves as an alternative meal option when rice or bread-based meals are not chosen. The carbohydrate amounts in the sandwich and chicken salad were 94.0 g and 59.3 g, respectively, compared to 90.8 g in the rice-based meal. Despite the differences in carbohydrate content and energy intake among these meals, we observed that the postprandial glycemic curves were influenced by the order of carbohydrate proportions in the meals. The reduced glycemic response observed in the sandwich and chicken salad meals can be attributed to the combined effects of protein and fat contents, which align with findings from previous studies [18,26]. Additionally, it may be affected by the lower GI of bread than white rice. Previous studies reported better glycemic response control with low-GI diets [15,27,28]. However, it is unlikely that healthy adults will exclusively consume all meals with low-GI diets or modify meals by adding protein and fats. Instead, it is more practical and informative to understand how individuals respond to meals with varying ranges of carbohydrate levels. 

We found that a chicken salad (carb 45%) showed no correlation between 14-day glycemic variability and glycemic control. Considering a study that a diet based on high-GI foods, including 50% of carbohydrates from foods with GI values > 70, increased glycemic variability [14], meal types with less than 45% carbs, such as chicken salad, can be used as major meal options for people who have a high response to carbohydrate-rich meals. Since everyone has their own food preferences, and no one wants to have the same meal type at every meal, it is important to have individualized diets. However, it is also crucial to take a meal-based approach rather than focusing on single food items when considering individualized diets. 

Healthy diets include a variety of foods with balance. In our study, we specifically tested a fruit bowl since fruit consumption has been linked to the health benefits of cardiovascular disease [29]. However, concerns have been raised regarding the high content of simple sugar in fruits. In this study, the fruit bowl consisted solely of fresh fruits, and we observed a rapid increase in PPGR in the early time, similar to the finding seen with fructose [8]. However, the PPGR at 2 h was similar to that of a chicken salad meal. This can be explained by the fact that while a fruit bowl contains relatively high dietary sugar, it also provides a considerable amount of dietary fiber. Dietary fiber has been reported to attenuate glycemic excursions [30], and previous studies also reported that fresh fruit consumption significantly lowered blood pressure and blood glucose levels [31], and whole-fruit consumption, not fruit juice, was associated with a reduced risk of obesity [32]. This indicates that a fruit bowl with fresh fruits can be a good option for meals. 

Carbohydrates are an essential macronutrient and a primary source of energy. While a wide range of carbohydrate intakes is acceptable, greater importance has been placed on consuming carbohydrates from whole-grain cereals, legumes, vegetables, and whole fruits to improve overall diet and carbohydrate quality [5,33]. Moreover, it is important to note that individual responses to carbohydrates can vary based on people’s physiology. Certain individuals might experience higher responses compared to others. Hence, individuals concerning carbohydrate intake are advised to find their own PPGR for carbohydrate-rich meals.

Another interesting finding of this study was that 14-day glycemic control or glycemic variability was not correlated with daily nutrient intake in 3-day dietary records. Traditionally, dietary intake based on 3-day food records reflects usual intake, and it is represented as the average daily intake. However, high carbohydrate intake in this study was related to neither a high CV nor high mean glucose. Rather, the iAUC of meals with above 50% of carbohydrates showed a strong correlation with glycemic variability or glycemic control. This indicates that meal-based strategies are more useful for improving glycemic control and glycemic variability. 

There are several limitations to this study. First, the small sample size does not allow the generalization of findings to other groups. Further studies are necessary to confirm our findings in a larger sample. Second, we did not examine the postprandial glycemic response to each meal beyond 2 h. Although a fruit bowl has the potential to decline to below fasting blood levels, no one reported any hunger or any hypoglycemic events. As our participants were apparently healthy without diabetes, it is also necessary to confirm results in adults with impaired glucose tolerance. Lastly, as dietary intake was assessed using the dietary record method prior to the intervention, it may not fully represent the participants’ usual intake during the intervention. Nevertheless, we ensured that the participants did not alter their habitual diet during the CGM active period. The average intake derived from 3-day dietary records is considered a useful estimate of usual dietary intake. However, further study is needed to confirm whether daily nutrient intake is associated with 14-day glycemic variability. Despite several limitations, this study is the first to demonstrate individual PPGRs to meal types with varying carbohydrate contents and their impact on glycemic variability. As studies on individual PPGRs to common meal types accumulate, they can be used for meal-based dietary guidance. 

## 5. Conclusions

In summary, our study found that the mean glucose excursion for mixed meals with varying carbohydrate levels corresponds to the proportions of carbohydrates in those meals. We also identified individuals who exhibited high responses in postprandial glycemic response. Furthermore, the 14-day glycemic variability was associated with the incremental AUC of the meals. To improve glycemic variability and control, it is crucial to understand the glycemic response to carbohydrate-rich meals and adopt a meal-based approach when planning diets. 

## Figures and Tables

**Table 1:** The food ingredients and nutrient composition of meals according to different carbohydrate levels.

Meal Type	Amount (g)	Glycemic Index	Energy (kcal)	Carb (g)	Sugars (g)	Fiber (g)	Protein (g)	Fat (g)	SFA (g)
Type A. Bibimbap									
Rice	210.0	72.0	305.2	70.5	1.2	2.5	4.4	0.6	0.2
Fried egg	34.0	0.0	66.9	1.3	0.0	0.0	5.1	4.6	1.3
Stir-fried beef	17.0	43.0	22.7	0.7	0.0	0.0	2.0	1.3	0.6
Seasoned vegetables	68.0	42.0	78.5	6.0	1.3	3.4	2.4	5.0	0.7
Stir-fried mushrooms	23.0	31.0	30.7	2.3	0.0	2.1	0.7	2.1	0.1
Red chili paste	15.0	21.0	35.1	7.8	0.1	0.8	0.5	0.2	0.0
Soybean paste soup	168.0	14.0	19.3	2.2	0.0	1.0	1.3	0.6	0.1
Sum	535.0	63.7	558.4	90.8	2.5	9.8	16.4	14.4	3.0
				(65.0%)			(11.8%)	(23.1%)	(0.1%)
Type B. Sandwich									
Ciabatta	120.0	61.0	358.2	65.8	0.0	2.7	12.4	5.0	0.7
Ham	60.0	0.0	72.3	1.2	0.0	0.0	12.4	2.0	0.8
Cheese	10.0	0.0	28.6	0.5	0.0	0.0	2.8	1.7	1.9
Lettuce	35.0	32.0	7.3	1.6	0.1	0.5	0.2	0.0	0.0
Tomato	50.0	23.0	11.4	2.1	0.6	1.3	0.5	0.1	0.0
Mustard source	10.0	32.0	31.5	4.0	0.0	0.7	0.6	1.5	0.1
Soybean milk	190.0	44.0	163.9	18.8	4.0	1.7	6.1	7.2	1.2
Sum	440.0	54.2	673.2	94.0	4.7	6.9	35.0	17.5	4.7
				(55.9%)			(20.8%)	(23.4%)	(0.1%)
Type C. Chicken salad									
Romaine	48.0	32.0	13.6	2.2	0.4	1.8	0.9	0.2	0.0
Asparagus	18.0	32.0	3.8	0.5	0.1	0.5	0.4	0.0	0.0
Tomato	40.0	23.0	11.7	2.4	0.8	0.8	0.4	0.1	0.0
Cranberry	10.0	45.0	34.9	8.6	3.4	0.6	0.0	0.1	0.0
Sweet pumpkin	60.0	64.0	45.7	9.3	1.2	3.1	1.0	0.5	0.1
Ricotta cheese	55.0	27.0	87.3	3.7	0.0	0.0	3.9	6.4	4.0
Chicken breast	85.0	0.0	102.6	0.0	0.0	0.0	23.9	0.8	1.2
Sweet potato, baked	65.0	70.0	121.1	29.2	0.0	2.4	0.8	0.1	0.0
Balsamic dressing	30.0	50.0	104.0	3.4	1.5	0.0	0.1	10.0	0.4
Sum	411.0	58.0	524.8	59.3	7.4	9.2	31.4	18.2	5.7
				(45.1%)			(23.9%)	(30.9%)	(0.1%)
Type D. Fruit bowl									
Apple	150.0	44.0	93.1	20.4	9.6	4.1	0.3	1.2	0.0
Orange	200.0	45.0	104.7	23.6	5.2	4.2	1.8	0.3	0.0
Banana	105.0	47.0	97.7	23.0	7.0	2.0	1.2	0.1	0.0
Green grape	70.0	54.0	50.3	12.2	5.6	1.2	0.2	0.0	0.0
Sum	525.0	46.7	345.8	79.2	27.4	11.5	3.5	1.6	0.1
				(91.7%)			(4.0%)	(4.2%)	(0.0%)

**Table 2:** The general characteristics of participants at baseline.

	Total *n* = 34	Men *n* = 9	Women *n* = 25	*p* Value ^1^
Basic characteristics				
Age (year) (mean ± SD)	22.5 ± 3.0	24.0 ± 1.7	22.0 ± 3.2	0.0174
Alcohol consumption, *n* (%)				0.7362
Yes	25 (73.5)	7 (20.6)	18 (52.9)	
No	9 (26.5)	2 (5.9)	7 (20.6)	
Current smoker, *n* (%)				0.0033
Yes	5 (14.7)	4 (11.8)	1 (2.9)	
No	29 (85.3)	5 (14.7)	24 (70.6)	
Physical activity, *n* (%)				0.0464
High	9 (26.5)	5 (14.7)	4 (11.8)	
Moderate	20 (58.8)	4 (11.8)	16 (47.1)	
Low	5 (14.7)	0 (0.0)	5 (14.7)	
Body measurement				
Body mass index (kg/m^2^)	22.8 ± 3.8	24.7 ± 4.0	22.1 ± 3.5	0.0725
Muscle mass (kg)	24.1 ± 6.4	33.0 ± 5.1	20.8 ± 2.8	<0.0001
Fat mass (kg)	19.0 ± 8.3	16.9 ± 8.4	19.7 ± 8.2	0.5981
Body fat (%)	29.7 ± 8.9	21.3 ± 8.3	32.8 ± 7.1	0.0034
Waist circumference (cm)	74.8 ± 10.1	83.5 ± 9.5	71.6 ± 8.5	0.0025
Nutrient intake				
Energy (kcal)	1492.9 ± 533.5	1770 ± 758.4	1393.1 ± 400.6	0.2116
Energy (% of EER)	65 ± 20.3	62.2 ± 22.7	66 ± 19.7	0.4823
Carbohydrate (%)	51.1 ± 7.7	52.3 ± 12.3	50.6 ± 5.5	0.2919
Protein (%)	17.1 ± 3.1	17.6 ± 3.4	16.9 ± 3	0.7253
Fat (%)	32.4 ± 7.9	30.5 ± 12.5	33.1 ± 5.7	0.2416
Sugars (%)	11.7 ± 4.6	9.4 ± 3.5	12.5 ± 4.7	0.0610
Saturated fat (%)	11.7 ± 3.9	11.2 ± 6.1	11.9 ± 2.8	0.5069

^1^ *p*-values for group comparisons according to Chi-square test for categorical variables and Wilcoxon rank-sum test for continuous variables.

**Table 3:** The correlation analysis of postprandial glycemic response to test meals and baseline characteristics with CGM outcomes.

	14-Day Glycemic Variability		14-Day Glycemic Control	
	Coefficient ^2^	(*p* Value)	Coefficient ^2^	(*p* Value)
Basic and body measurement				
Age (year)	0.2371	(0.1991)	0.2486	(0.1775)
Body weight (kg)	−0.1919	(0.3010)	−0.0285	(0.8790)
Muscle mass (kg)	−0.0775	(0.6785)	0.1396	(0.4539)
Body fat (%)	−0.1366	(0.4637)	−0.1923	(0.3001)
Postprandial glucose 2 h after test meals				
Glucose 75 g	0.5547	(0.0012)	0.4969	(0.0045)
Type A. Rice and soup	0.3325	(0.0676)	0.6399	(0.0001)
Type B. Sandwich and soymilk	0.5039	(0.0038)	0.5445	(0.0015)
Type C. Chicken salad	0.0759	(0.6847)	0.4192	(0.0189)
Type D. Fruit bowl	0.5088	(0.0035)	0.3586	(0.0476)
Incremental area under curve after test meals				
Glucose 75 g	0.6363	(0.0001)	0.4057	(0.0236)
Type A. Rice and soup	0.5785	(0.0007)	0.4537	(0.0104)
Type B. Sandwich and soymilk	0.6842	(<0.0001)	−0.0158	(0.9327)
Type C. Chicken salad	0.2428	(0.1882)	0.2322	(0.2088)
Type D. Fruit bowl	0.6023	(0.0003)	0.2204	(0.2334)
Daily dietary intake from 3 days of dietary records				
Energy (kcal)	−0.0367	(0.8448)	0.0373	(0.8423)
Carbohydrate (%)	0.0314	(0.8670)	0.0019	(0.9917)
Protein (%)	0.1418	(0.4466)	0.0571	(0.7602)
Fat (%)	−0.1514	(0.4162)	−0.0223	(0.9052)
Average fasting blood glucose ^1^	0.0942	(0.6143)	0.7568	(<0.0001)
CGM mean glucose (mg/dL)	0.2406	(0.1922)		

Glycemic variability and control were defined as coefficient of variation and mean glucose during CGM active period. ^1^ Average fasting blood glucose was calculated as the mean of fasting blood glucose from five different days. ^2^ Spearman correlation coefficient was presented with *p* value in parenthesis after adjusting for sex.

## Data Availability

All datasets generated or analyzed during this study are available from the corresponding author upon reasonable request.

## Supplementary Materials

The following supporting information can be downloaded at https://www.mdpi.com/article/10.3390/nu15163571/s1, Figure S1: Flow chart for selection of study subjects; Figure S2: The blood glucose levels measured using CGM and SMBG; Table S1: The degree of fullness and meal duration according to the meal type.
