# Supplementary material for: Individual Postprandial Glycemic Responses to Meal Types by Different Carbohydrate Levels and Their Associations with Glycemic Variability Using Continuous Glucose Monitoring
Source: Nutrients. 2023 Aug 13;15(16):3571. doi: 10.3390/nu15163571 (PMC10459284; doi:10.3390/nu15163571)
Supplement: Supplementary file 1 [file nutrients-15-03571-s001.zip › nutrients-2550474-supplementary.pdf]

**Supplementary Figures and Tables for Song et al., "Individual postprandial glycemic responses to meal types by different carbohydrate levels and their associations with glycemic variability using continuous glucose monitoring"**

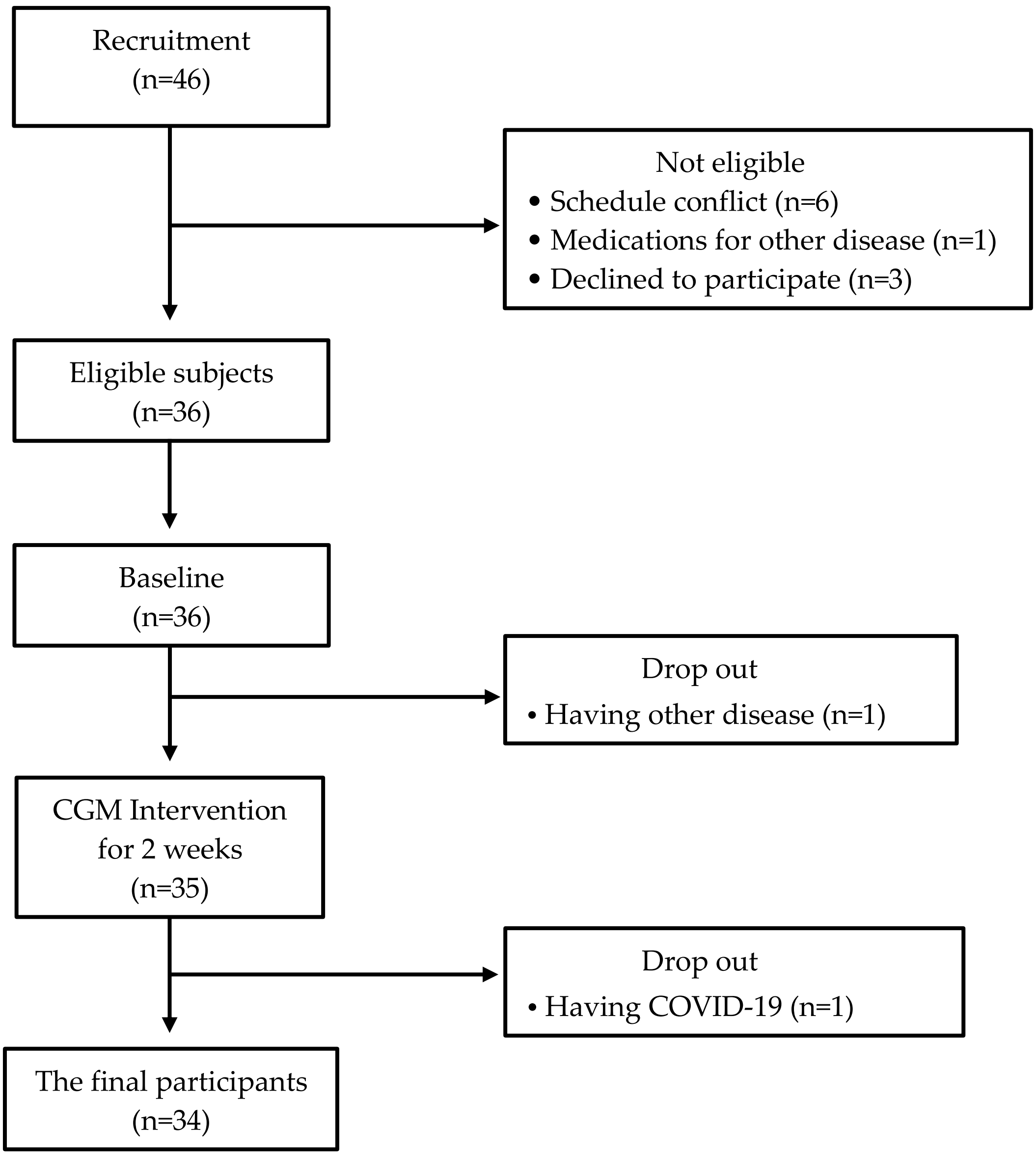


**Figure S1**. Flow chart for selection of study subjects

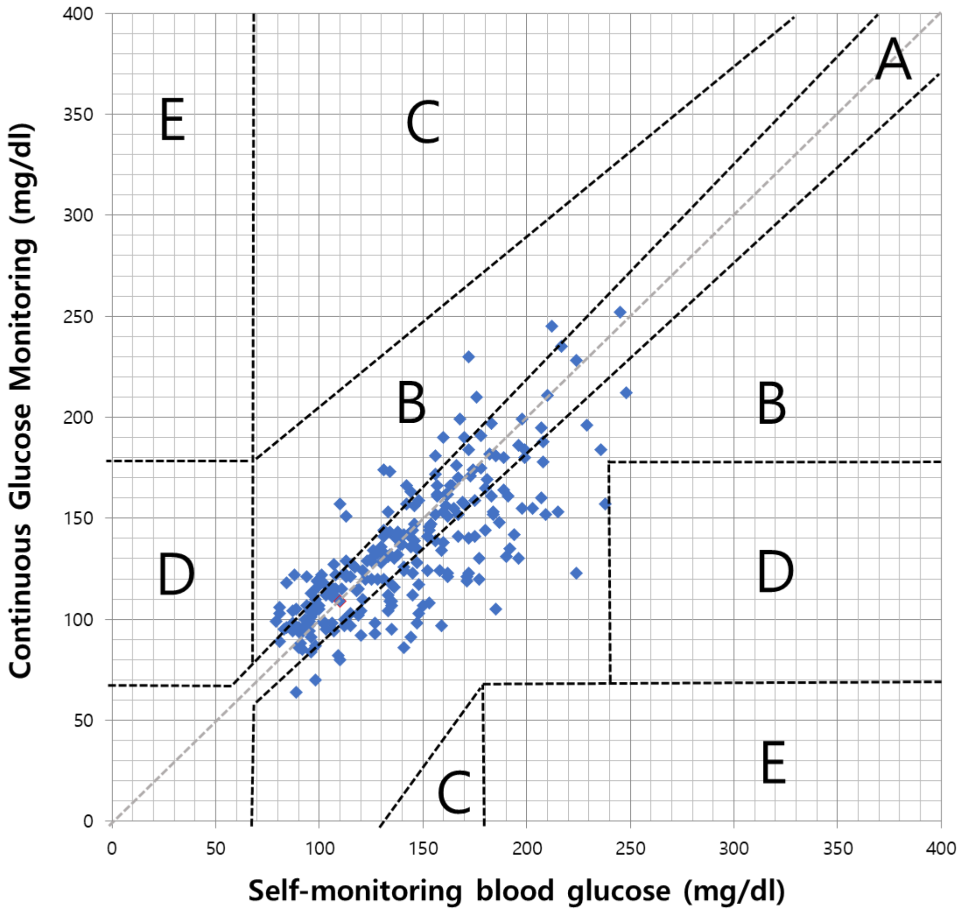


**Supplementary Figure 2.** The blood glucose levels measured by CGM and SMBG

**Supplementary Table 1.** The degree of fullness and meal duration according to the meal type

| | Meal type | | | | P-value[1] |
|---|---|---|---|---|---|
| | A (n=34) | B (n=33) | C (n=34) | D (n=34) | |
| Meal duration (min) | 16.6 ± 6.3[b] | 15.2 ± 5.9[b] | 21.8 ± 6.2[a] | 20.7 ± 5.6[a] | **<.0001** |
| (Interquartile range) | (11.0 - 22.0) | (11.0 - 20.0) | (16.0 - 27.0) | (17.0 - 25.0) | |
| Fullness, n (%) | | | | | 0.1852 |
| Hungry | 1 (2.9) | 3 (9.1) | 4 (11.8) | 4 (11.8) | |
| Neutral | 1 (2.9) | 8 (24.2) | 7 (20.6) | 6 (17.7) | |
| Satisfied | 7 (20.6) | 12 (36.4) | 6 (17.7) | 8 (23.5) | |
| Full | 16 (47.1) | 5 (15.2) | 10 (29.4) | 8 (23.5) | |
| Very full | 9 (26.5) | 5 (15.2) | 7 (20.6) | 8 (23.5) | |

[1] P-values are calculated by ANOVA and Chi-square test
